# A new approach of inducing proprioceptive illusion by transcutaneous electrical stimulation

**DOI:** 10.1186/s12984-021-00870-y

**Published:** 2021-05-03

**Authors:** Rohit Rangwani, Hangue Park

**Affiliations:** Department of Electrical and Computer Engineering, Texas A&M University, College Station, TX 77843 USA

**Keywords:** Transcutaneous electrical stimulation, Proprioception, Proprioceptive illusion, Kinesthetic illusion, Proprioceptive modulation, Arm matching experiment, Pinocchio illusion

## Abstract

**Background:**

Neurotraumas or neurodegenerative diseases often result in proprioceptive deficits, which makes it challenging for the nervous system to adapt to the compromised sensorimotor conditions. Also, in human machine interactions, such as prosthesis control and teleoperation, proprioceptive mismatch limits accuracy and intuitiveness of controlling active joints in robotic agents. To address these proprioceptive deficits, several invasive and non-invasive approaches like vibration, electrical nerve stimulation, and skin stretch have been introduced. However, proprioceptive modulation is still challenging as the current solutions have limitations in terms of effectiveness, usability, and consistency. In this paper, we propose a new way of modulating proprioception using transcutaneous electrical stimulation. We hypothesized that transcutaneous electrical stimulation on elbow flexor muscles will induce illusion of elbow joint extension.

**Method:**

Eight healthy human subjects participated in the study to test the hypothesis. Transcutaneous electrodes were placed on different locations targeting elbow flexor muscles on human subjects and experiments were conducted to identify the best locations for electrode placement, and best electrical stimulation parameters, to maximize induced proprioceptive effect. Arm matching experiments and Pinocchio illusion test were performed for quantitative and qualitative analysis of the observed effects. One-way repeated ANOVA test was performed on the data collected in arm matching experiment for statistical analysis.

**Results:**

We identified the best location for transcutaneous electrodes to induce the proprioceptive illusion, as one electrode on the muscle belly of biceps brachii short head and the other on the distal myotendinous junction of brachioradialis. The results for arm-matching and Pinocchio illusion tests showed that transcutaneous electrical stimulation using identified electrode location and electrical stimulation parameters evoked the illusion of elbow joint extension for all eight subjects, which supports our hypothesis. On average, subjects reported 6.81° angular illusion of elbow joint extension in arm-matching tests and nose elongated to 1.78 × height in Pinocchio illusion test.

**Conclusions:**

Transcutaneous electrical stimulation, applied between the the synergistic elbow flexor muscles, consistently modulated elbow joint proprioception with the illusion of elbow joint extension, which has immense potential to be translated into various real-world applications, including neuroprosthesis, rehabilitation, teleoperation, mixed reality, and etc.

## Background

Proprioception is a perception or awareness of the position or movement of one’s own body [1]. It includes both the sense of body position in space and the sense of force applied to each joint, as the somatosensory cortex processes a combination of evoked action potentials from sensory receptors in muscles, tendon, and skin [1–6]. Proprioception also includes dynamic perception of the body, i.e., sense of body movement, along with the stationary perception, and it is called as dynamic proprioception or kinesthesia [1, 4]. Proprioception plays an important role in our daily lives, as it provides us sensory information necessary to complete motor tasks with minimal reliance on vision. For example, we can walk on the street or step on car pedals without looking at our legs and feet. Indeed, we rely on proprioception for most of our motor activities throughout the day without even recognizing it. Proprioceptive deficit can cause serious deficits in motor control, as seen in several human and animal experiments [7, 8]. Proprioceptive deficit in the arm muscles can easily disrupt the inter-joint coordination and degrade following control accuracy in arm reaching for human subject [7]. In animal locomotion study, a local deficit of proprioception in one of the leg muscles induced the cats to select inefficient locomotor strategy [8].

Proprioceptive deficit is generally caused by injuries or diseases [9–12] which affect central and peripheral nervous system. These deficits affect motor learning and are critical in progression of rehabilitation [13, 14]. We also observe similar deficits in man-made interface likes prosthetic limbs and telerobotic systems, where proprioceptive feedback of end effectors is not available to users. Even in the research field, artificially-generated proprioceptive feedback has been demonstrated in a very limited way, while artificially-generated tactile feedback has made some strides recently [15–20]. To address the proprioceptive deficit in the above applications, especially for the fine and sophisticated motor tasks [21–23], potential of neuromodulation has been actively investigated.

Invasive methods like intraneural stimulation techniques using high-resolution electrodes [24–31] have shown great potential in providing users with proprioceptive feedback. However, the amount of proprioceptive modulation is hardly consistent over time and across subjects [28, 30] and the chronic usage needs further validation before adaptation [32]. Further, the entry barrier for users, in accepting the surgical procedures and associated risks, makes these invasive approaches less attractive for non-desperate applications like rehabilitation in chronic conditions, human–robot interfaces, etc. Non-invasive methods using mechanical vibration have also been investigated to modulate proprioception, and successfully induced proprioceptive illusions [1–4]. Vibration onto the tendon, myotendinous junction, or muscle belly elicited illusions of stretch/extension of the associated muscle [33–35]. However, the vibration-induced illusions are still hard to be elicited in a consistent manner [35–40], because this approach is sensitive to the location of vibrators, vibration parameters, quality of contact, and fatigue of target muscle [33]. The direction of vibration-induced illusions sometimes can be even opposite [21, 35] and application of subthreshold vibrations with random frequencies can augment joint proprioception for both extension and flexion [38]. Skin stretch is another non-invasive approach of providing proprioceptive information [41, 42], as the skin stretch is an important contributing factor to the perception of joint extension/flexion. This approach can also be used together with other proprioceptive modulations as a compensatory approach. However, skin stretch system is hard to be implemented, because rotational skin-stretch devices need to be well attached onto the skin with proper friction [41].

Multiple indirect approaches have also been investigated for providing proprioceptive information without directly modulating proprioceptive feedback. For example, tactile augmentation and proximity feedback have been used to deliver proprioceptive information [23, 43]. As tactile feedback and proprioception usually work together as an ensemble to deliver the static and dynamic information of the body parts, tactile augmentation or haptic feedback can help the nervous system to make a better decision under the lack of proprioception [41]. However, the efficacy of this ensemble drops significantly if the motor task does not involve any physical interaction with the object. Proximity feedback has been introduced recently to enjoy the power of tactile augmentation at limb movement without any physical interaction [23], but the quality of information and intuitiveness is limited.

**Fig. 1:**
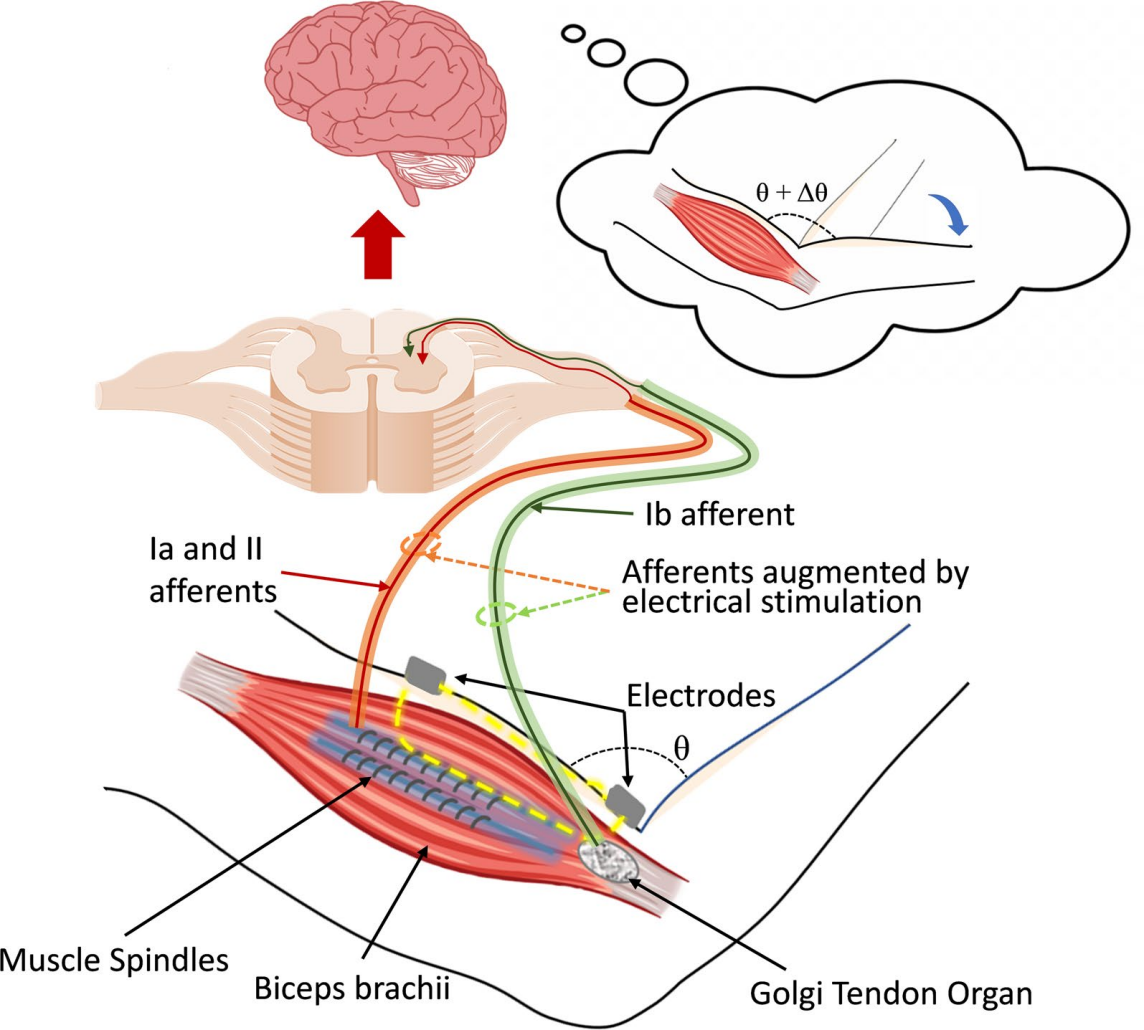
Concept figure. Transcutaneous electrical stimulation using surface electrodes targeting biceps brachii muscle to augment muscle spindle afferents to induce proprioceptive illusion of arm extension

In the presented study, we tested a novel approach of transcutaneous electrical stimulation for proprioceptive modulation. Success of invasive approaches of electrical nerve stimulation [24–31] and transcutaneous muscle vibration, in generating and modulating the proprioception, suggest that transcutaneous electrical stimulation might be also an effective way to generate or modulate the proprioception. Till now, transcutaneous electrical stimulation has been mostly targeted to provide tactile feedback or sensory cue [44, 45], mainly because of the limited accessibility to the nerves from the skin. However, we expect that, similar to the approach of mechanical vibration, transcutaneous electrical stimulation can also excite the muscle spindle or Golgi tendon organ [46, 47] to induce proprioceptive illusions, as described in Fig. 1. Although transcutaneous electrical stimulation will induce mixed perception of tactile feedback and proprioception, by stimulating skin receptors or cutaneous nerves [48, 49] as well as muscle spindle or Golgi tendon organ [46, 47], this non-invasive electrical stimulation approach has so many advantages over its invasive counterpart, such as high user acceptability with easy and safe non-invasive interface. It also provides advantages over muscle vibration approach, by providing small form factor, robust contact interface with surface electrode, and high controllability with electrical signals.

As a proof of concept, we hypothesize that transcutaneous electrical stimulation on biceps brachii and brachioradialis, with appropriate stimulation parameters at appropriate location, will augment the perception of the elbow extension. Because biceps brachii short head and brachioradialis [47] are synergistic muscles for elbow flexion, the augmented afferent feedback from the muscle spindles is expected to augment the perception of elbow extension. In this study, we developed and validated the efficacy of transcutaneous electrical stimulation on inducing proprioceptive illusion of the elbow joint.

## Methods

### Human subject recruitment

All experiments were performed adhering to relevant guidelines and regulations, in accordance with the procedure described in the protocol approved by Institutional Review Board, Texas A&M University (IRB2018-1583D). Eight healthy human subjects in age 22–35 (with average of 26.1), one female and 7 males participated in the study. All subjects were right-handed. Subjects with neurological disorder, cognitive impairment, upper limb deformity, and any known allergic problem to skin adhesive were excluded from the study. All subjects provided their informed consent for the experimentation according to the approved IRB protocol.

### System implementation

A biphasic voltage-controlled electrical stimulator was designed for providing transcutaneous electrical stimulation to subjects. Voltage-controlled stimulator provides an effective and easy way to generate electrical stimuli. The system consisted of a microcontroller (Particle Photon with STM32 ARM Cortex M3) to generate input pulse-width-modulated (PWM) waveforms followed by a level shifter to increase the voltage level and convert it into biphasic square-wave output. Microcontroller was programmed to generate biphasic square-wave output with frequency ranging over 0–10 kHz and duty factor over 0–100%, according to the operator input. A small signal NPN transistor (MMBT3904, On Semiconductor, AZ) was used for voltage level shifting to generate the actual voltage stimulus ranging over 3–30 V. The biphasic voltage stimuli were generated by an H-bridge circuit, composed of CMOS n-channel and p-channel FET (field effect transistor) pairs (CD4007UE, Texas Instrument, TX) [48]. The system was powered by a rechargeable Li-Po battery. A step-up voltage converter was used for converting 3–4 V from Li-Po battery to high voltage levels up to 30 V.

Custom designed transcutaneous gel electrodes were used to deliver the electrical stimulus to the skin over the target muscle. The custom designed electrodes were made using the reusable self-adhesive electrode. These reusable electrodes were customized in size and multithreaded connecting wires were stacked on top using silver conductive epoxy [23, 48]. Electrodes were customized with a small footprint (approx. 1.2 × 0.8 cm^2^), to ensure high localization of the electrical stimulation and identify the appropriate electrode locations with maximum effect. The self-adhesive hydrogel was pasted on the electrodes and additional latex-free adhesive tape was taped over the electrodes, to ensure stable contact of electrodes onto the skin during arm movements.

Two gyroscope sensors (MPU9250) were strapped onto both left and right forearms, one for each arm, to record the angular data for both elbow joints. An elastic strap with Velcro was used to fasten the gyroscope sensors on subjects’ arms to allow for minimum perturbations. The sensitivity scale factor of 131 (LSB)/°/s and a full-scale range of ± 250°/s is used for the selected gyroscope sensors. The gyroscope data was digitized using a built-in 16-bit ADC in MPU9250 to provide high-resolution data, and then sampled at 10 Hz by the microcontroller. Gyroscopes were calibrated every time before the experiment, for data integrity. The gyroscope data was delivered to the microcontroller via SPI interface and saved to the computer via USB interface. As gyroscope provides the derivative of an angle, the desired angle values were calculated by integrating the gyroscope data over time.

### Experiment procedure

#### Parameter selection for electrical stimulus

For the all experiments, we selected biphasic square-wave electrical stimulus for charge balancing. 50% duty factor and 10-ms inter-pulse interval (i.e., 100 Hz) were also selected as default stimulation parameters, based on previous successful experiments that showed the effect of electrical stimulation on proprioceptive modulation [28, 47]. The amplitude range of the stimulation was determined per subject based on subjective perception, between perception and discomfort thresholds. As the stimulation was continuously applied during the given “stimulation-on” duration, determined by each experimental condition, we didn’t specify the stimulus train duration and Inter-stimulus interval.

#### Experiment I: identification of electrode placement

**Fig. 2:**
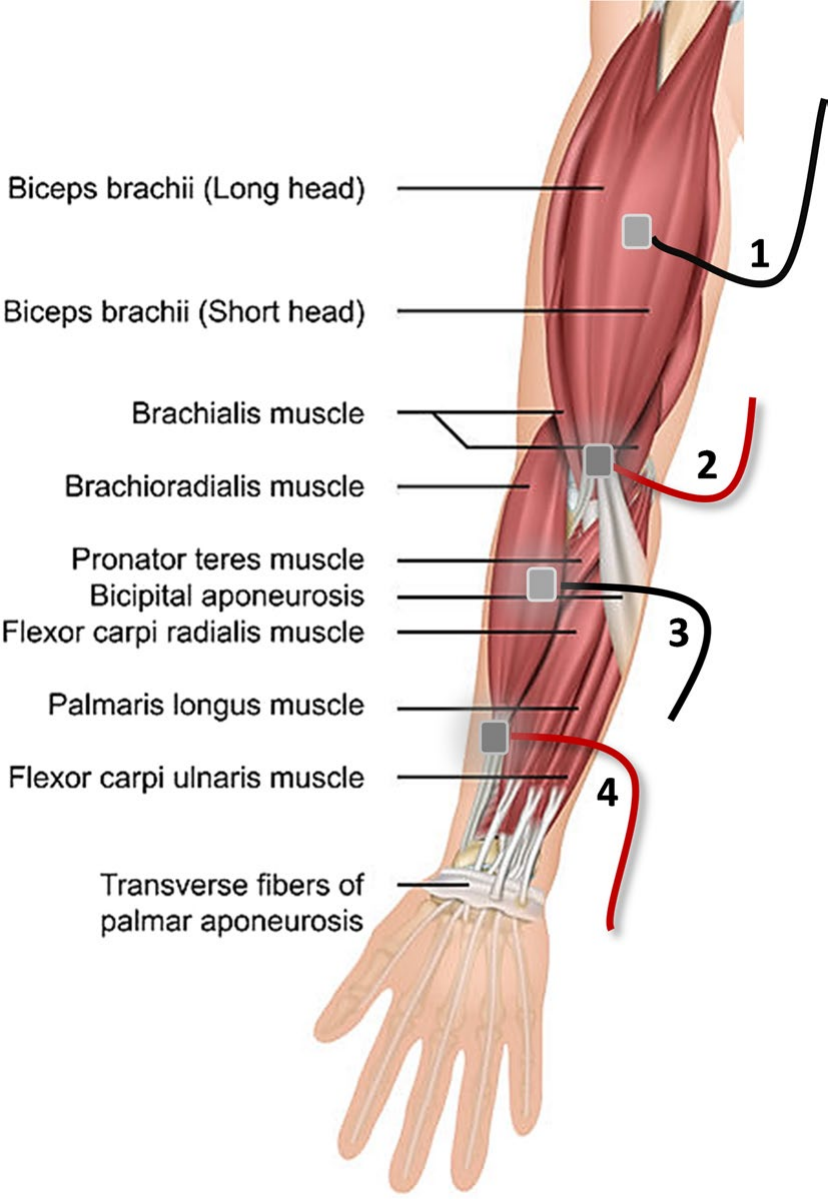
Representation of the identified relevant electrode locations. Representation of the relevant electrode locations identified in the first experiment, (1) muscle belly of biceps brachii short head, (2) distal myotendinous junction of brachii short head, (3) muscle belly of brachioradialis, and (4) distal myotendinous junction of brachioradialis (* the image of the arm muscles was adapted from the image downloaded from bilderriese © 123RF.com)

The first experiment was designed to identify the location of electrodes for transcutaneous electrical stimulation. We selected four different locations of bipolar electrodes, on biceps brachii short head and brachioradialis. Spindle afferent feedback from these two muscles, across the elbow joint, contributes to the perception of the elbow joint angle [50]. As it is hard to determine the accessibility of transcutaneous current to reach the muscle spindle via the muscle belly and the myotendinous junction areas, we targeted both of those areas for two synergistic elbow flexor muscles. Figure 2 shows all four identified electrode locations and combination of these four locations resulted in 10 different locations for a pair of electrodes.

With a pair of electrodes placed on selected locations, we set the stimulation voltage amplitude as 5 V. No subject reported any sensation at 5 V stimulation voltage. The voltage was then gradually increased and the voltage amplitude at which subjects first reported some paresthesia or any stimulation-based sensation was recorded as perception threshold. The voltage amplitude was further increased to a level until subjects reported any discomfort. We set the stimulation voltage right below this discomfort level where subjects were comfortable to be stimulated for long duration. Once the required voltage for proprioceptive illusion was identified, the electrical stimulation was turned on and subjects were asked to report the subjective feeling of arm flexion or extension (i.e., proprioceptive illusion) by 1–5 subjective rating while providing stimulation for 5-s duration. This was repeated two times in series. The 1–5 scale was defined to evaluate the intensity of proprioceptive illusion, as 1 represents no illusion and 5 represents very strong illusion (1—nothing, 2—barely perceivable, 3—perceivable, 4—strong, 5—very strong).

Stimulation frequency was fixed at 100 Hz for the Exp. I and the duty factor of the biphasic stimulus was fixed as 50%, according to the previously successful parameters for evoking electrotactile feedback [51]. Subjects were asked to maintain elbow joint angle at 90˚, where 180˚ means fully extended elbow joint. The selected voltage level and the corresponding subjective rating of proprioceptive illusion were recorded. Based on this subjective rating, we intended to select the best electrode pair location to be used for the following experiments to test proprioceptive illusion. Note that the experiment I was executed only for the first three subjects. It is because the location for the strongest proprioceptive illusion was clear and consistent for the first three subjects. To minimize potential aftereffect of the stimulation, experiments for the other five subjects used the selected electrode location based on the result of the first three subjects.

#### Experiment II: characterization of electrical stimulation parameters

The second experiment was designed to identify the best amplitude and frequency of electrical stimulation for the maximal proprioceptive illusion, with positioning electrodes on the best location found at the first experiment. Subjects were asked to place their arm at rest with their elbow firmly placed onto the 90° armrest for reference [21]. Although the armrest stays at the location during the experiment to guide the joint angle of the right arm, subjects were asked to actively maintain the guided elbow joint angle and not to rest on the armrest. In other words, subjects were barely touching it instead of resting on it. Subjects were then blindfolded, and voltage level was gradually increased. Subjects were asked to report when the electrotactile feedback, generally described as tingling, started to set the perception threshold (*V*_*th*_). The voltage level was further increased, until the subject reported any discomfort. The maximum stimulation voltage right below the discomfort range was set as the maximum voltage level (*V*_*max*_). The amplitude of the stimulation used for the experiment was determined per subject based on subjects’ report of the maximal proprioceptive illusion, by slowly increasing the amplitude from *V*_*th*_ to *V*_*max*_. Note that, standard psychophysics methods were not used for measurement of the thresholds and the amplitude of the stimulation used, which is a limitation of this study.

We also identified the appropriate frequency of electrical stimulation for the proprioceptive modulation, with the same procedure of arm resting and blindfold. For this part, the voltage was fixed to the *V*_*max*_ found earlier with the frequency fixed at 100 Hz. Subjects were asked to report the effect when the stimulation frequency was changed from 100 Hz. Subjects were provided stimulation with a set of frequencies of 30, 100, 300, 1000, and 3000 (Hz) and asked to rate the proprioceptive illusion from 1–5 for each frequency.

#### Experiment III: arm matching experiment

**Fig. 3:**
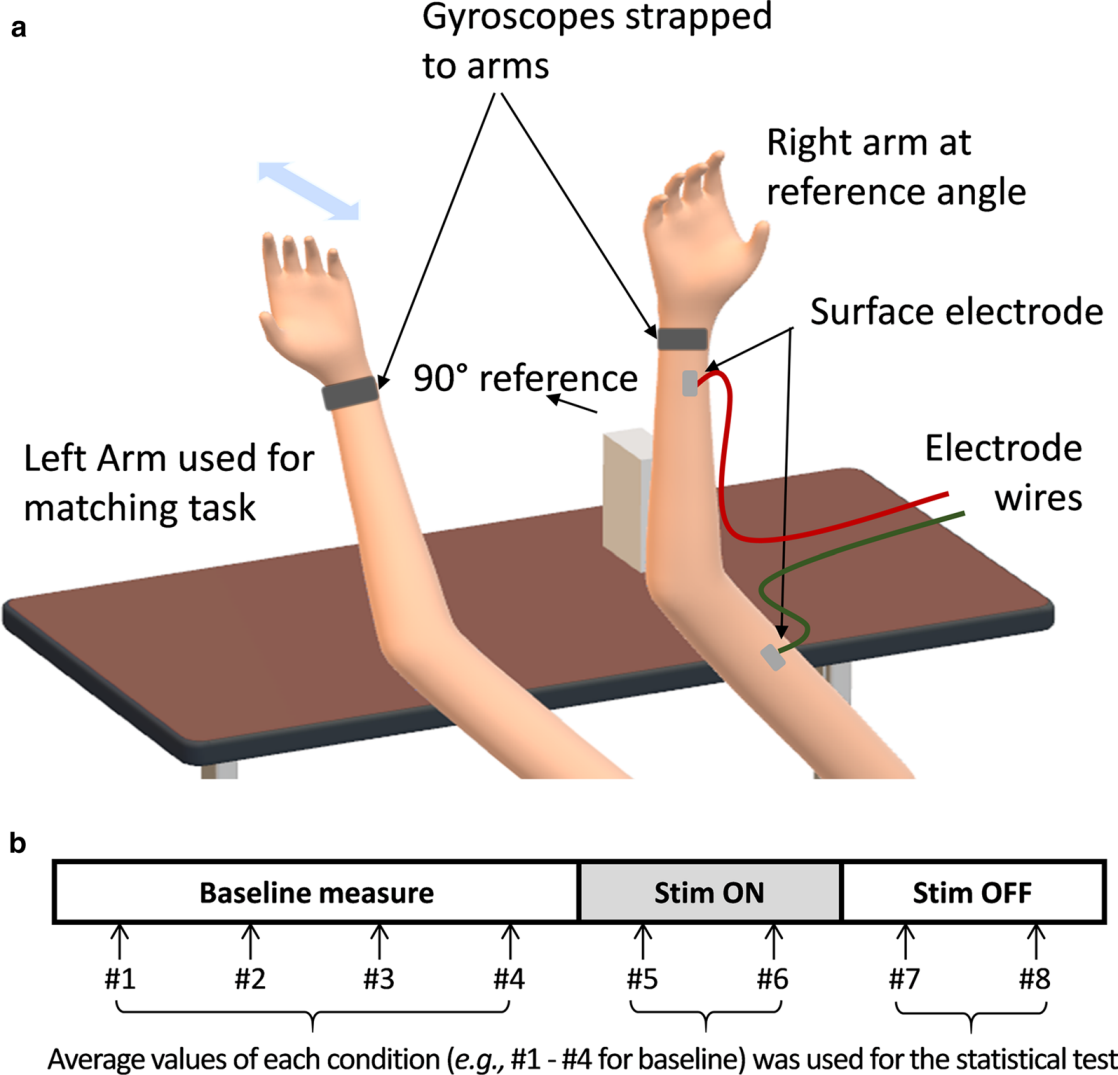
Proprioceptive illusion intensity and electrical stimulation parameters. **a** Intensity of proprioceptive illusion for 10 pairs of electrode locations, obtained with 3 initial subjects (Bi: Biceps brachii, Br: Brachioradialis, M: Muscle belly, and T: Distal myoTendinous junction; e.g., BiM: Biceps brachii muscle belly); **b** Intensity of proprioceptive illusion for different stimulation frequencies with the best electrode location (BiM-BrT), averaged for all subjects; **c** Perception threshold (*Vth*) and maximum applicable voltage (*Vmax*) for each subject, with the best electrode location (BiM-BrT); and (d) percentage voltage activation with respect to Vth (Vmax/ Vth*100) for each subject. Values in (**b**) are represented as mean ± standard error (SEM)

The third experiment was designed to quantify the angular displacement induced by transcutaneous electrical stimulation. Arm-matching test was selected for quantification of the illusory flexion/extension of the elbow joint, as it has been proved as a reliable way to evaluate proprioceptive illusion in prior works [1, 4, 5]. As shown in Fig. 3, subjects were asked to place their arm at rest with their elbow firmly placed onto the 90° or 135° armrest for reference. Subjects were clearly instructed to actively maintain the guided elbow joint angle and not to rest on the armrest. To ensure consistent muscle pre-conditioning, the subjects were instructed to bring their right arm close to shoulder before maintaining it at reference angle at start of each trial. Note that, such muscle conditioning/thixotropy maximizes the illusory effect of elbow extension, as shown in previous vibration-based proprioceptive modulation studies [1, 33].

**Fig. 4:**
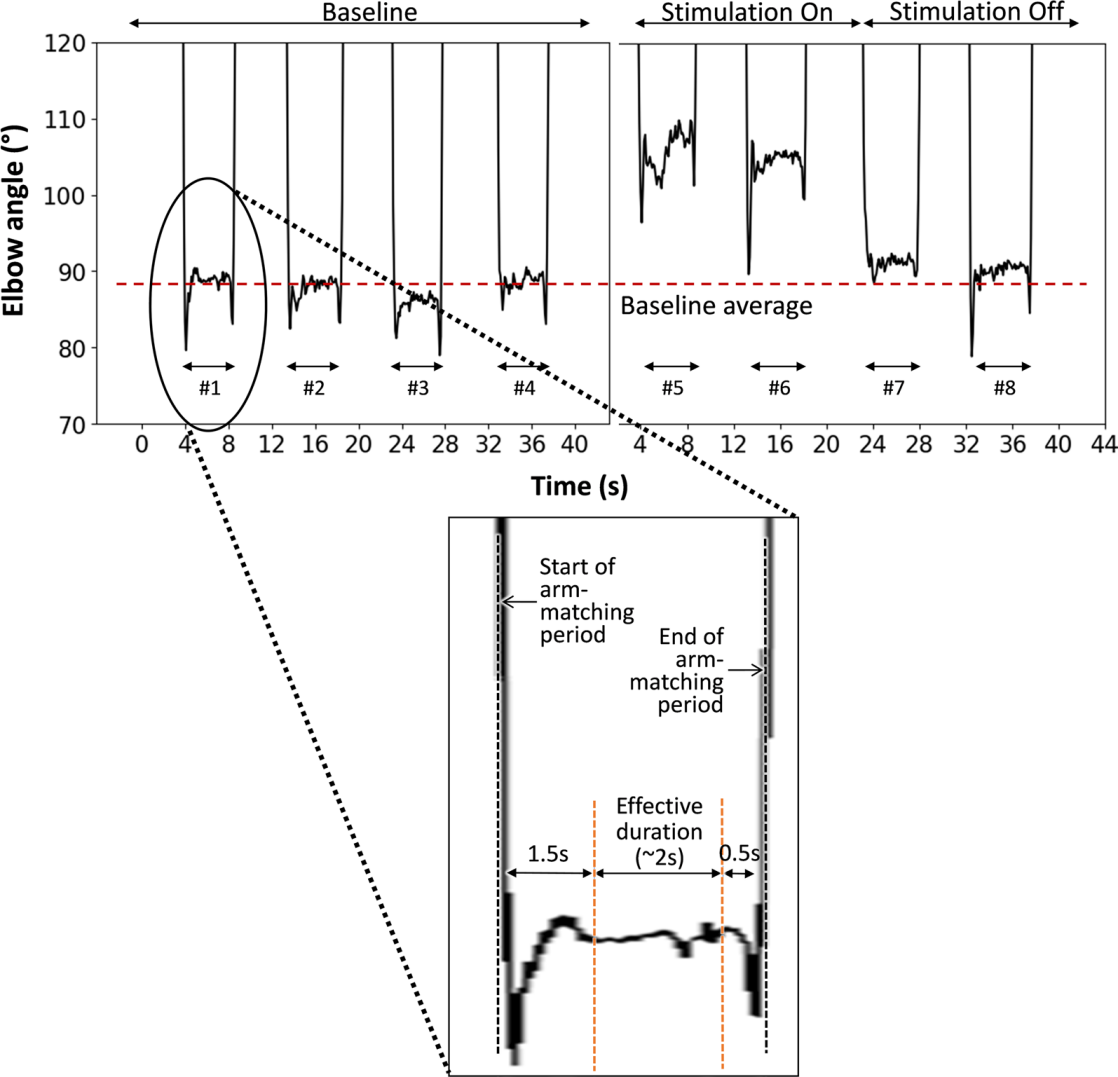
Proprioceptive illusion data in arm matching experiment and Pinocchio illusion experiment for all 8 subjects. **a** Individual data of left elbow joint angle in arm matching experiment with 135° right elbow joint angle (reference); **b** Individual data of left elbow joint angle in arm matching experiment with 90° right elbow joint angle (reference), **c** Subjective perception of nose elongation when the stimulation is turned on (black circle) and subjective perception of nose shrink when the stimulation is turned off (white circle), in Pinocchio illusion experiment; **d** Average left elbow joint angle in arm matching experiment with 135° right elbow joint angle (reference); **e** Average left elbow joint angle in arm matching experiment with 90° right elbow joint angle (reference); **f** Perception of nose height change from the previous height; **g** Standard deviation of right elbow joint angle while the stimulation was applied; **h** Net change of left elbow joint angle; and **i** Relative change of left elbow joint angle with respect to the initial angle. *Represents statistical significance (p < 0.05) by one-way repeated ANOVA test with 95% confidence level. Overall value in the very left side of (**c**), and values in (**d**), (**e**), (**f**), (**h**), (**i**) are represented as mean ± standard error (SEM)

Subjects were also asked to relax their arm muscles and keep their right arm stationary. Subjects were blindfolded during the experiment to avoid any bias from the visual feedback. Each session was composed of baseline measurement, stimulation test, and aftereffect test. First, for baseline measure, subjects were asked to do arm matching without any stimulation provided. Initially, subjects were asked to keep their right arm stationary at specified reference angle and keep their left arm in fully extended posture. When they hear the audio command, “Flex”, they were asked to use their left arm to match the right arm elbow joint angle. When they hear the audio command “Go back”, they were asked to return their left arm to the fully extended posture resting their arm on the desk. A constant time delay of 4 s was provided between each audio command for subjects to complete the required arm movement and keep the elbow joint angle at that angle. This arm-matching test was repeated for four times in series to ensure that the baseline value was consistent. The gyroscope data for this arm matching sequence was saved to a computer. Second, to measure the effect of electrical stimulation, electrical stimulation was applied on the best identified location with best identified parameters. Subjects were then asked to move their left arm to match the elbow joint angle between the left and right elbow joint. According to the audio commands, the arm-matching test was repeated for two times in series. Third, to measure the aftereffect of electrical stimulation, the arm-matching test was repeated for two times in series, as shown in Fig. 4. The gyroscope data for stimulation on/off sequences was saved to a computer. The same procedure (2 consecutive sessions) was conducted for two different reference joint angles: 90° and 135°, with a minimum of 1-min interval between sessions for each reference joint angle. The session order was counterbalanced among subjects with randomization (in a random order, 4 subjects conducted 90° first and the other 4 subjects conducted 135° first). In summary, two sessions for two different reference target elbow joint angles were conducted for all the subjects following the above-mentioned procedure. Within the single session, arm matching test was repeated for four times to measure a baseline, repeated for another two times with stimulation applied, and repeated for another two times after stimulation was turned off (see Fig. 3b).

At the end of the arm matching experiment, we collected subjective descriptions about the proprioceptive illusion, from all eight subjects, to get more insight about the principle of the proprioceptive illusion. In addition, we also collected subjective description of the accompanying unnatural sensation for all eight subjects as we know that transcutaneous electrical stimulation often evokes unnatural sensation, referred to as tingling or paresthesia.

#### Experiment IV: Pinocchio illusion experiment

The fourth experiment was designed to confirm that the induced illusion is proprioceptive in nature. Pinocchio illusion experiment has been used in past [52, 53], to establish and understand the proprioceptive illusion. This classical experiment involves the subject touching their nose with a fingertip of the same arm on which the stimulus for inducing proprioceptive illusion is applied. Subjects were blindfolded for this experiment and we used the best electrode location and stimulation parameters identified in Exps. I and II, to induce the maximum proprioceptive illusion while minimizing potential bias caused by visual feedback. Based on the direction of illusion, subjects perceived their nose tip elongated or shrunk.

**Fig. 5:**
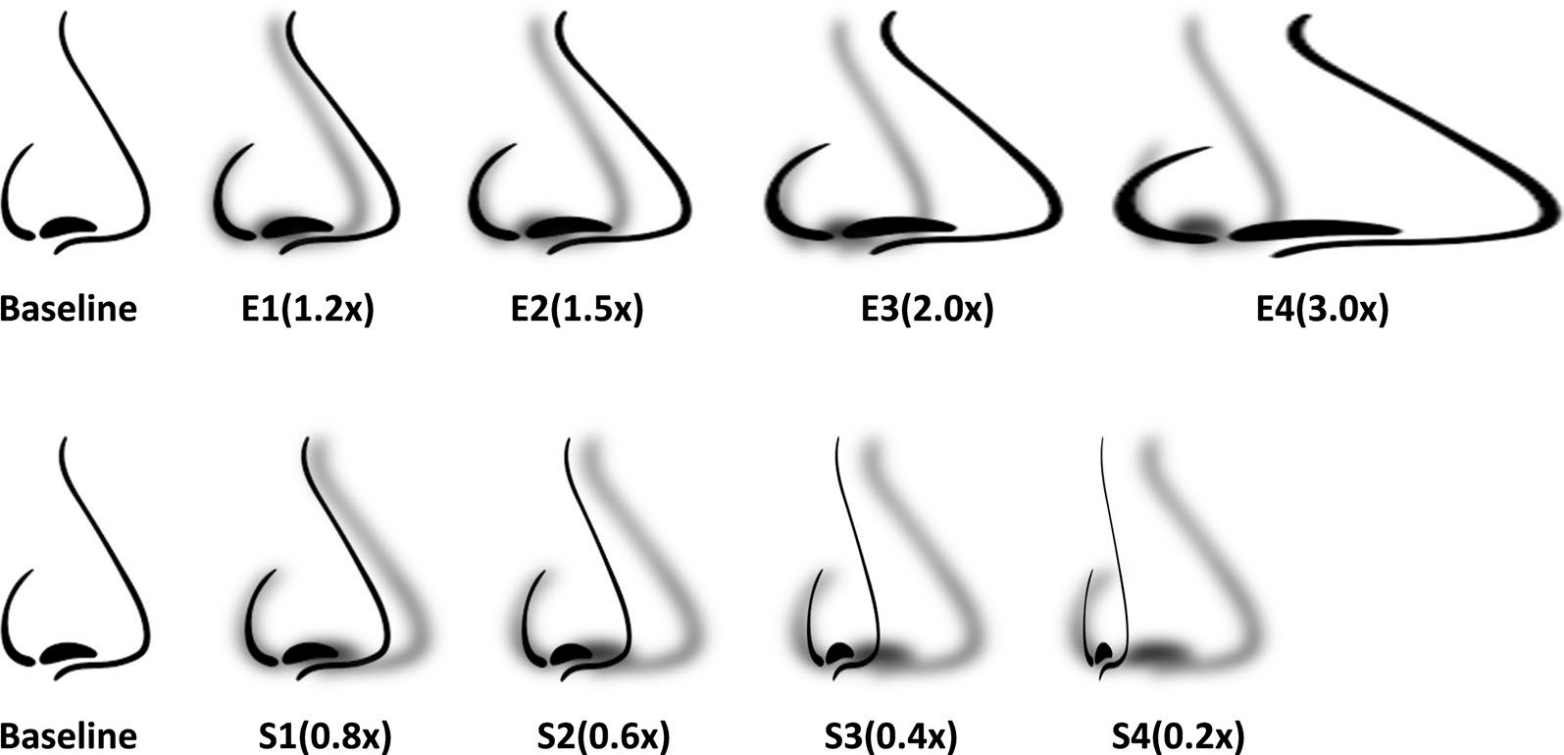
Pinocchio illusion experiment nose illustrations. Illustrations of nose for subjects to select from for Pinocchio illusion experiment for illusion of nose elongation (corresponding to illusion of arm extension; E1–E4) and for illusion of nose shrinkage (corresponding to illusion of arm flexion; S1–S4)

Subjects were instructed to touch their nose tip using their right index fingertip after being blindfolded. The biphasic electrical stimulation was provided for a duration of 10 s on their right arm with electrodes placed on previously identified locations. Subjects were asked to keep their index fingertip on the nose tip for 10 s after the stimulation was turned off, to report any aftereffect. Subjects were asked to select the pictorial representation of nose (on a scale of 1–5 for both elongation and shrinkage) from a series of nose representations that best describes their feeling (as shown in Fig. 5).

#### Data analysis and statistics

For the subjects’ data in the arm-matching test, elbow angles of the left arm were calculated as the average value within effective duration of each measure. To define the effective duration, we first defined both the start and end of the arm-matching period, as the times when the elbow angle is crossing the value 30° larger than the target elbow angle (165° for 135° target and 120° for 90° target), as shown in Fig. 4. We then excluded 1.5 s from the start of the arm-matching period, to minimize the transient response. We additionally excluded 0.5 s at the end of the arm-matching period, as shown in Fig. 4, to minimize the effect of undershoot before movement. As a result, the effective duration for each measure is around 2 s, and the average of data for this effective duration was calculated and used. As shown in Fig. 4, in each subject, the average of the first four arm-matching periods (#1 to #4) was used as a baseline value before applying stimulation, the average of the next two measures (#5, #6) was used as a value for evaluating stimulation effect, and the average of the last two measures (#7, #8) was used as a value for evaluating stimulation aftereffect.

For statistical analysis of the data, one-way repeated ANOVA test was performed at the 95% confidence level, to conduct a robust statistical test not to be falsified by any small difference in variances. We selected one-way repeated ANOVA with having just time (before, during, and after stimulation) as an independent variable, instead of two-way ANOVA with having time and the reference elbow joint angle as two independent variables. It is because different reference elbow joint angles may set different conditions of muscle spindle and the result for one reference joint angle may be under- or overrepresented by the result for the other reference joint angle. To verify that the data satisfies the prerequisites for the ANOVA test, we tested normality of data distribution using the Kolmogorov–Smirnov test of normality. All datasets satisfied the condition of p > 0.05 and normality could be assumed. We also applied Bonferroni correction, and then used p < 0.05 as the condition for statistical significance. IBM SPSS Statistics was used as a statistical software.

## Results

### Experiment I

#### Stimulation across the two synergistic elbow flexor muscles was found as most effective in evoking proprioceptive illusion of the elbow joint angle

**Fig. 6:**
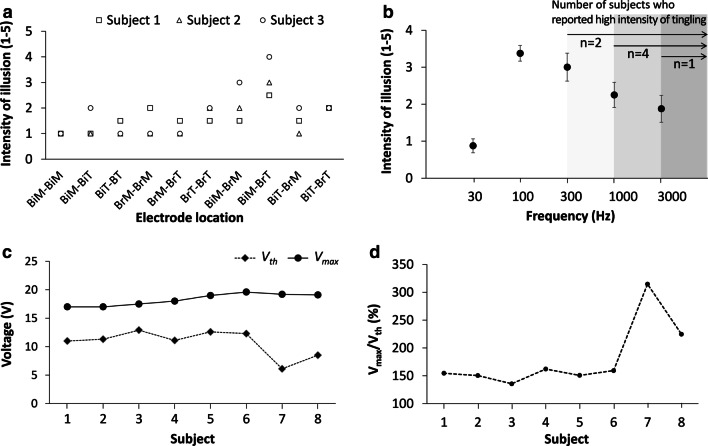
Arm matching experiment setup. **a** Schematic representation for arm-matching experiment and **b** detailed experimental procedure of the arm-matching experiment. Right arm elbow angle is maintained at a specified reference angle with help of armrests and subjects use their left arm to match the left elbow angle to the perceived right arm angle, with or without electrical stimulation applied. Gyroscopes were strapped onto both the arms, to measure the elbow joint angles

In the experiment for identifying the best location of electrodes, subjects were asked to report the strength of proprioceptive illusion when electrical stimulation was applied across the pairs of four different locations on the elbow flexor muscles of the right arm (ten different combinations; see Fig. 2). Figure 6a shows the proprioceptive illusion for ten combinations of selected electrode locations, reported by the first three subjects, on a scale of 1–5. The result suggests that the electrode pair, with one electrode on the belly of biceps brachii short head and another on the distal myotendinous junction of brachioradialis, could consistently evoke the maximum proprioceptive illusion of the elbow joint angle for each subject.

### Experiment II

#### In terms of stimulation amplitude, proprioceptive illusion was strongest with stimulation voltage just below the discomfort threshold, in the range of 15–20 V

In the experiment for the characterization of perception evoked by transcutaneous electrical stimulation, stimulation frequency was fixed at 100 Hz in accordance with previous studies on electrotactile feedback [54] for identifying the optimal stimulation amplitudes (*i.e.,* absolute value of positive/negative peak voltage for biphasic square-wave stimulus). All subjects reported proprioceptive illusion above a perception threshold (*V*_*th*_), and the proprioceptive illusion became stronger as the stimulation amplitude increased, before subjects felt discomfort along with strong tingling and paresthesia (see Table 1). The strongest proprioceptive illusion was observed at the maximum stimulation amplitude just below the stimulation amplitude evoking discomfort (*V*_*max*_; discomfort threshold), according to the subjects’ report of perception. Figure 6c shows the *V*_*th*_ and *V*_*max*_ for all eight subjects. The optimal stimulation voltages were found as 15–20 V for all subjects, as shown in Fig. 6c. In Fig. 6d, the voltage level providing strongest proprioceptive illusion was represented as a ratio with respect to *V*_*th*_, which is *V*_*max*_*/V*_th_*100.

**Table 1:** Subjective description of the proprioceptive illusion effect for all subjects

Subject	Intensity^*^	Description of proprioceptive illusion	Description of unnatural sensation
#1	3	Forearm is pushed down by external force	Tingling, vibrating, paresthesia
#2	3	Forearm is moving downward Vibrating/scratching on the skin	Tingling, vibrating, pulsing, paresthesia
#3	3	Forearm is moving downward Vibrating on the skin	Tingling, vibrating
#4	3	Forearm is pushed down by external force	Tingling
#5	3	Forearm is pushed down by external weight Vibrating/scratching on the skin	Tingling, itchiness
#6	4	Forearm is moving downward	Tingling
#7	4	Forearm is pushed down by external force	Tingling
#8	3	Forearm is moving downward	Tingling, pulsing

Intensity and subjects’ description of the proprioceptive illusion, along with subjects’ description of the unnatural electrotactile sensation accompanied with the proprioceptive illusion

*Intensity of proprioceptive illusion on 1–5 scale (1—nothing, 2-—barely perceivable, 3—perceivable, 4—strong, 5—very strong)

#### In terms of stimulation frequency, 100 Hz was most effective in evoking proprioceptive illusion with minimal paresthesia

After the optimal voltage values were identified in the above experiment, we also identified the best suitable frequency for electrical stimulation to induce the maximum proprioceptive illusions, using the stimulation voltage identified for each subject. In the range of 30–3000 Hz, 100 Hz provided the strongest proprioceptive illusion with an average of 3.38 at 1–5 subjective scale (see footnote of Table 1 for details of the scale), as shown in Fig. 6b, and therefore 100 Hz was selected as stimulation frequency for the following experiments. Subjects commonly reported that a tingling sensation accompanied the proprioceptive illusion, and the level of tingling became stronger as frequency increases, which may have lowered the effective level of proprioceptive illusion. Therefore, we also identified the frequency range where subjects reported high intensity of tingling, with the number of subjects corresponding to the range, as depicted by grey shading of Fig. 6b. One subject did not report high intensity of tingling till 3000 Hz.

#### Proprioceptive illusion was accompanied with unnatural tingling sensation

All subjects reported unnatural electrotactile sensation accompanied the proprioceptive illusion, and this electrotactile sensation was described as tingling, vibrating, pulsing, paresthesia, and itchiness. Table 1 summarizes the unnatural sensation accompanying proprioceptive illusion, reported by each subject.

### Experiment III

#### Transcutaneous electrical stimulation on biceps brachii and brachioradialis evoked proprioceptive illusion of elbow joint extension, with no aftereffect

**Fig. 7:**
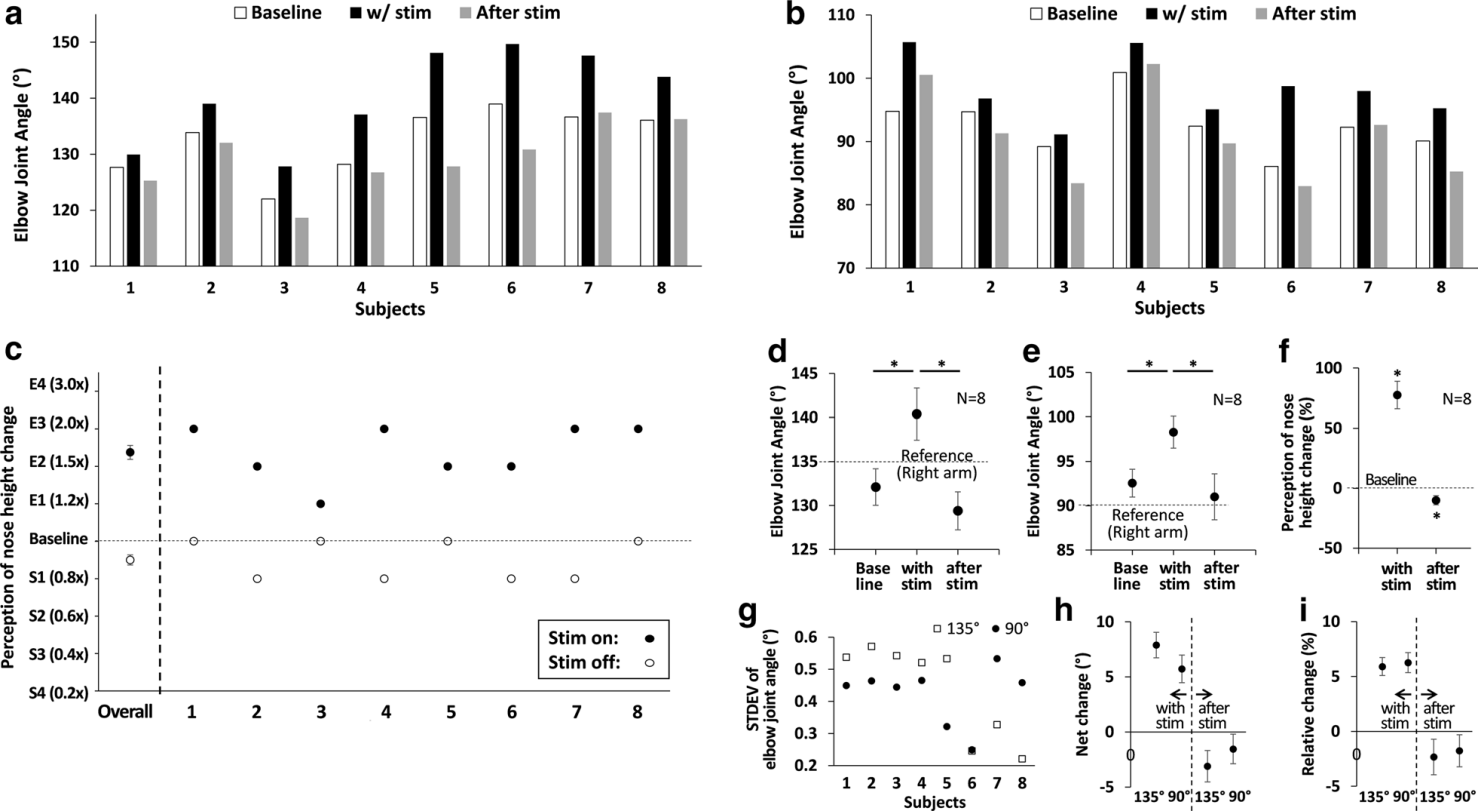
Data processing and analysis representation. The graphical representation of the exemplary temporal change of the left elbow joint angle, during the arm matching task with right elbow joint at reference angle of 90°. Horizontal dashed line in graph represents the average of the baseline measures of the left elbow angle, which was used as a reference to evaluate the extent of proprioceptive illusion. Bottom window, magnified from the first waveform, shows how we minimized potential errors during the transition phase in calculation of the elbow joint angle

Transcutaneous electrical stimulation on biceps brachii and brachioradialis was effective in generating the proprioceptive illusion of elbow joint extension, as left elbow joint angle was increased for both 135° and 90° reference angles (p = 0.002 and F(2,6) = 20.8 for 135° reference angle and p = 0.015 and F(2,6) = 9.16 for 90° reference angle). Figures 7d and 7e show the left elbow joint angle before any stimulation was applied (Baseline), when the stimulation was turned on (Stim.), and after stimulation was turned off (Aftereffect), for 135° and 90° reference angles, for all eight subjects. The data show that stimulation caused 7.89° and 5.73° angular displacement on average (illusion of elbow joint extension), for 135° and 90° reference angles, respectively. The strength of proprioceptive illusion was reported as 3.25 on average at the scale of 1–5 (5 is strongest; see footnote of Table 1 for details of the scale). As shown in Figs. 7a and b, all eight subjects consistently reported proprioceptive illusion of their elbow joint angle in the direction of elbow extension. Subjects’ description of the proprioceptive illusion can be largely categorized as three: “Forearm is moving downward”, “Weight or external force is pushing the forearm down”, and “Something is vibrating or scratching on the skin”, as in Table 1. When the stimulation was turned off, left elbow joint angle was decreased for both 135° and 90° reference angles (p = 0.003 and F(2,6) = 17.8 for 135° reference angle and p = 0.004 and F(2,6) = 15.9 for 90° reference angle). No aftereffect was detected, as the baseline values and the values after stimulation were not different for both 135° and 90° reference angles. Also, as shown in Fig. 7g, the standard deviation for the right arm elbow angle during the stimulation for both 135° and 90° reference angles are less than 0.6° for all the subjects indicating the subjects’ right arm were stable during the stimulation period. We also calculated and represented net change and relative change of the replicated elbow joint angle, as in Figs. 7h and i. The net changes of the replicated elbow joint angle were 7.89 ± 1.16° and 5.73 ± 1.43° for 135° and 90° reference angles, respectively. The relative changes of the replicated elbow joint angle were 5.90 ± 0.82% and 6.26 ± 1.62% for 135° and 90° reference angles, respectively.

### Experiment IV

#### Pinocchio illusion was experienced by all subjects, in the direction of elongation of nose

All subjects reported Pinocchio illusion upon application of the electrical stimulation with the electrodes placed one at muscle belly of biceps brachii and the other at distal myotendinous junction of the brachioradialis. Subjects reported illusion of elongation or shrinkage of nose, with stimulation-on and stimulation-off, respectively. Subjects were asked to select the most appropriate graphical representation of nose-height change among the ones shown in Fig. 5. As in Fig. 7c, all subjects reported nose elongation when stimulation was turned on (to 1.78 × on average, in the middle of E2 and E3). When the stimulation was turned off, subjects reported nose shrinkage or no change (to 0.9 × on average, in the middle of Baseline and S1). The change in perception of the nose height was statistically significant for the nose elongation when the stimulation was turned on (p < 0.001 and F(2,6) = 45.8). Subjects also felt nose shrinkage (from the normal nose height) when the stimulation was turned off (p = 0.019 and F(2,6) = 8.24), which suggests aftereffect of the stimulation.

## Discussion

### Transcutaneous electrical stimulation induced proprioceptive illusion in the direction of augmenting muscle spindle afferent feedback

Transcutaneous electrical stimulation resulted in the proprioceptive illusion in the direction of augmenting the muscle spindle afferent feedback. In our experiment, stimulation across biceps brachii short head and brachioradialis (synergistic elbow flexors) evoked proprioceptive illusion in the direction of elbow extension. This result suggests that the stimulation augmented the muscle spindle afferent feedback from both biceps brachii short head and brachioradialis. We expect that the identified electrode locations on the biceps belly and tendinous area of brachioradialis provided a proper current pathway to activate muscle spindle of both muscles.

This result agrees with the general observations of vibration-induced proprioceptive illusions, causing stretch/extension illusion of the muscle on which the vibration is applied [1–4, 33]. In case of the vibration-induced proprioceptive illusion, vibrating elbow flexor muscle resulted in the illusion of elbow joint extension and vibrating elbow extensor muscles resulted in the illusion of elbow joint flexion. We expect that the transcutaneous electrical stimulation works in similar way to the vibration-induced proprioceptive illusion. However, note that, other studies have reported an opposite effect of vibration-induced proprioceptive illusion, as suppressing the effect of muscle spindle afferent feedback based on the stimulation parameters [21, 35–39, 55, 56]. In the follow-up studies, it would be important to investigate the consistency of the electrically-induced proprioceptive illusion.

### Stimulating muscle spindles in two synergistic elbow flexors together seems critical in proprioceptive illusion by transcutaneous electrical stimulation

As seen in prior works, the location of application was a critical factor for inducing proprioceptive illusion by applying vibration or electrical stimulus on muscles and tendons [1, 2, 31, 33, 34, 44]. In the examples of vibrotactile proprioceptive illusion applied on muscle, vibration on one agonist muscle (e.g., biceps) for elbow joint flexion was enough to evoke proprioceptive illusion in the direction of elbow joint extension. However, based on our experiment, we found that transcutaneous electrical stimulation applied across two synergistic elbow flexor muscles together evoked the strongest proprioceptive illusion of elbow joint extension. Figure 6a shows that the electrode pair across two synergistic elbow flexor muscles, biceps brachii and brachioradialis, provided much stronger proprioceptive illusion than stimulating one of the two muscles. We speculate that it is because elbow extension usually causes change in length of both muscles, and contradictory information between two muscle spindles may suppress the gain of the proprioceptive feedback.

### The intensity of proprioceptive illusion depends on the voltage level of transcutaneous electrical stimulation, as well as the site of stimulation

We found that the intensity of the proprioceptive illusion was the strongest at the level just below the discomfort threshold, consistently for all eight subjects. Our interpretation of this phenomenon is that transcutaneous electrical stimulation activates voltage-gated ion channels in dermis as well as the ones in sensory fibers, and therefore evokes discomfort on the skin. Indeed, all eight subjects reported tingling as a main unnatural electrotactile sensation potentially causing discomfort at high intensity (see Table 1). There is also a possibility that involuntary muscle activation by stimulation contributed to the proprioceptive illusion. Although we selected the stimulation intensity as the value below the discomfort threshold, we cannot exclude the possibility of muscle activation. Therefore, we checked the standard deviation of the right elbow joint angle, and minimal standard deviation (< 0.6°) suggests that the right arm was not moved involuntary or unconsciously by the stimulation (Fig. 7g). We also investigated the voltage level providing strongest proprioceptive illusion, with respect to the voltage level of the perception threshold. As shown in Fig. 6d, the voltage level providing the strongest proprioceptive illusion was 50–200% higher than the voltage level of the perception threshold.

### The stimulation frequency on proprioceptive illusion seems not as critical as vibration-induced illusion, but needs to be further investigated

Vibration-induced proprioceptive illusion has been reported as being highly dependent on the frequency. According to the current understanding, vibration can selectively activate muscle spindle afferents according to the vibration frequency. For example, vibration with higher frequencies activates secondary (II) spindle afferent, and vibration with lower frequencies activates primary (Ia) spindle afferents [33, 40]. Cordo et al. reported that the direction of proprioceptive illusion was opposite according to the frequency of vibration, even at the same subjects [39]. Our previous work also suggests that the proprioceptive illusion could be evoked with very narrow range of vibration frequency [21]. For electrical stimulation, on the other hand, frequency seems not as critical as in the case of vibration in evoking the proprioceptive illusion. Although we found the optimal stimulation frequency as 100 Hz to maximize the proprioceptive illusion, other frequencies such as 300 Hz and 1000 Hz also evoked the proprioceptive illusion in the same direction with comparable intensity. However, there is a possibility that induced paresthesia may have overrode proprioceptive illusion, considering that a few subjects reported tingling sensation at frequency of 300 Hz or higher. In the follow-up study, we will investigate further about the optimal frequency with lowering the stimulation intensity and minimizing the tingling sensation. The combined effect between stimulation frequency and amplitude should be further investigated too, as vibration studies showed that both vibration amplitudes and frequency are co-dependent on evoking the proprioceptive illusion [33, 39, 40].

### We cannot exclude the potential effect of cutaneous electrotactile feedback on proprioceptive illusion, which should be investigated in depth in future studies

Previous studies on skin stretch showed that stretch of the skin near the joint evoked proprioceptive illusion, which was further amplified when skin stretch was used along with the vibration [1, 38, 51, 57]. These results suggest that cutaneous electrotactile feedback may contribute to the proprioceptive illusion [53], by potential coordination with the muscle spindle afferent feedback. However, as electrotactile feedback evokes unnatural cutaneous sensations (e.g., tingling, vibrating, pulsing, paresthesia) instead of skin stretch or contraction, it is hard to interpret the contribution of cutaneous electrotactile feedback on proprioceptive illusion. In the future studies, the effect of cutaneous electrotactile feedback and its coordination with the muscle spindle afferent feedback, on proprioceptive illusion, needs to be investigated in depth.

### Follow-up research is needed to investigate the effect of other factors on proprioceptive illusion

Studies on vibration-induced illusion suggest that both static and dynamic states of associated muscle (e.g., muscle pre-conditioning, initial joint angle, muscle thixotropy) have strong effects on proprioceptive illusions [1, 33, 39]. Therefore, we expect that the effect of transcutaneous electrical stimulation on proprioceptive illusion will also depend on the static and dynamic states of muscle spindle. Indeed, to apply the proprioceptive illusion using transcutaneous electrical stimulation in the real-life applications, it is important to understand the effect of both the past and current states of the muscles, along with its dynamics, on the proprioceptive illusion. We cannot also exclude the possibility that visual feedback of the associated muscles and joints may have affected the proprioceptive illusion, as multisensory processing with both visual and proprioceptive feedback is known to affect the sensory perception [58]. Note that, although the subjects were blindfolded during the experiment, they could see the initial arm angle when they positioned the arm to the armrest. Further, we cannot exclude the psychological factors influencing the proprioceptive illusion. As subjects were asked about illusory feelings of elbow flexion/extension in the first and/or second experiments, subject may have unconsciously adjusted their responses in experiments three and four to match those expectations. Future studies are needed to investigate the effects of static and dynamic states of associated muscles, the effect of visual feedback, and the psychological effect to match the expectations of questionnaires, on the proprioceptive illusion.

We also observed that relative increase in the replicated elbow joint angle at arm matching test was not different between the one at 135° elbow joint angle and the one at 90° elbow joint angle (5.90 ± 0.82% and 6.26 ± 1.62% for 135° and 90° reference angles, respectively) (p = 0.846). Absolute increase of the replicated elbow joint angle at arm matching test was not different either between the two elbow joint angles (p = 0.26). Perhaps different length of the sarcomere did not affect the sensitivity of muscle spindle to the electrical stimulation. Note that the change in sensitivity of muscle spindle have been reported in past studies where the static and dynamic state of muscle and muscle thixotropy affected proprioception [1–4, 33]. It would be important to further investigate the effect of the elbow joint angle on the intensity of proprioceptive illusion evoked by transcutaneous electrical stimulation.

### Transcutaneous electrical stimulation has advantages over the vibration-induced proprioceptive modulation, in terms of latency, consistency, and implementation

Transcutaneous electrical stimulation has several advantages over the vibration-induced proprioceptive illusion, which is currently the most widely accepted non-invasive method for inducing proprioceptive illusion. First, the latency issue observed in vibration-induced proprioceptive illusion [1, 32] can be addressed by using the transcutaneous electrical stimulation. All subjects reported that the transcutaneous electrical stimulation caused proprioceptive illusion with minimal latency that they were not able to recognize. Second, the consistency of proprioceptive illusion can be improved by employing the transcutaneous electrical stimulation. Note that the biggest challenge for the vibration-induced proprioceptive illusion is the variation of the effect [1, 32]. Research studies in the past, using vibration, found that vibration did not induce proprioceptive illusion for part of the subjects [1, 32, 48, 56, 59]. Fuentes et al. reported that 10–20% of subjects did not feel proprioceptive illusion by the vibration [55] and Roll et al. also assumed the limited efficacy and restricted the subjects as previously successful participants who reported proprioceptive illusion by vibratory intervention [59]. We observed that the effect of transcutaneous electrical stimulation was consistent for all eight subjects who participated in the experiment, and the effect was consistent per subjects over the two sessions with two reference elbow joint angles (see Figs. 7a–c). However, note that, this study was conducted with a limited number of subjects (8) and sessions per subject (2), and therefore the inter- and intra-subject variability needs to be tested further in the future studies. Third, electrical stimulation allows for easy and simple system implementation, compared to the vibration-based approach. Fixation of the vibrator motor on a specific location of the arm is not easy because of the inherent properties of mechanical vibration resulting in mechanical deviation from the targeted location, and vibration reaching neighboring muscle groups. Also, the bulkiness of the mechanical vibration system makes it hard to implement the whole system as a small wearable device. On the other hand, electrical stimulation based approach can result in an easy-to-design, small-sized, highly localized, and consistent system without any mechanical deviation. Such electrical system will minimally disturb the natural arm and hand movements, and allow easy translation of such approach to real-world applications.

### No aftereffect in proprioceptive illusion could be another advantage of transcutaneous electrical stimulation for real-time proprioceptive modulation

Based on statistical tests regarding the results of arm matching experiment in both 90° and 135° joint angles (Figs. 7d and e), we concluded that there is no stimulation aftereffect (i.e., no difference between the baseline value and the value after the stimulation is turned off). In other words, proprioceptive illusion, initially generated by turning on the stimulation, could be cancelled without lasting effect. This feature of no aftereffect would be beneficial for modulating proprioception in real time, to evoke sequential proprioceptive illusion. The effect of stimulation on proprioceptive illusion will not be affected by the history of stimulation. Note that, results of Pinocchio illusion experiment (Fig. 7f) were not considered for the discussion of stimulation aftereffect, as there is no way to directly compare between the baseline value and the value after the stimulation is turned off.

## Conclusions

This study tested the novel approach of transcutaneous electrical stimulation to induce proprioceptive illusion on perceiving the elbow joint angle. Transcutaneous electrical stimulation on the synergistic elbow flexor muscles could evoke proprioceptive illusion in the direction of augmenting the spindle afferent feedback. The observation and results strongly suggest that transcutaneous electrical stimulation is not just a viable option for proprioceptive modulation but a strong candidate to enhance the user-acceptability and consistency of proprioceptive modulation. However, for practical use of this new approach, there are still many challenges to be addressed. We need to further clarify operating principles of transcutaneous electrical stimulation in inducing proprioceptive illusion, such as the contribution of cutaneous component of electrotactile feedback, the effect of static and dynamic states of associated muscle, the effect of visual feedback and multisensory processing, and the involvement of muscle spindle and Golgi tendon organ. Upon the robust establishment of effective parameters of transcutaneous electrical stimulation and understanding of its operating principle, on inducing proprioceptive illusion, the transcutaneous electrical stimulation has immense potential to be applied in proprioceptive modulation for neuroprosthesis, teleoperations, neurorehabilitation, and virtual reality. Another important limitation of this study is that all subjects were healthy and able-bodied. For this approach to be applied to people in need, having disability and/or sensorimotor deficits, follow-up studies are needed to show the efficacy of this approach on target population.

## Data Availability

The datasets generated during and analyzed during the current study are available from the corresponding author on reasonable request.

## Abbreviations

PWM: Pulse width modulation
FET: Field-effect transistor
IRB: Institutional Review Board
